# The First Complete Mitochondrial Genomes for the Genus *Dianema* (Siluriformes: Callichthyidae): *Dianema longibarbis* and *D. urostriatum*

**DOI:** 10.3390/genes16030355

**Published:** 2025-03-20

**Authors:** Seong Duk Do, Jae-Sung Rhee

**Affiliations:** 1 Department of Marine Science, College of Natural Science, Incheon National University, Incheon 22012, Republic of Korea; 2 Research Institute of Basic Sciences, Incheon National University, Incheon 22012, Republic of Korea; 3 Yellow Sea Research Institute, Incheon 22012, Republic of Korea

**Keywords:** mitogenome, Callichthyinae, *Dianema*, phylogenetic analysis

## Abstract

Background/Objectives: To date, no information is available on the complete mitochondrial genome of the genus *Dianema* (Siluriformes: Callichthyidae), a callichthyid catfish. In this study, we report on two complete mitochondrial genome sequences of *Dianema longibarbis* Cope, 1872, and *Dianema urostriatum* Miranda Ribeiro, 1912, the only two recognized species within the genus *Dianema*. Methods: DNA sequencing was performed using the HiSeq platform to obtain their complete mitogenomes. To confirm phylogenetic distance, two phylogenetic trees were established using maximum-likelihood and Bayesian inference methods with all concatenated protein-coding sequences (PCGs) and two ribosomal RNA (rRNA) genes from the *D. longibarbis* and *D. urostriatum* mitogenomes, along with 32 mitogenomes retrieved from Siluriformes. Results: The complete mitogenomes of *D. longibarbis* and *D. urostriatum* are 16,493 and 16,495 base pairs in length, respectively. Their nucleotide compositions are 31.79% A, 27.53% T, 25.86% C, and 14.82% G for *D. longibarbis*, and 31.69% A, 27.04% T, 26.36% C, and 14.91% G for *D. urostriatum*. Both mitogenomes contain 13 PCGs, 22 transfer RNA (tRNA) genes, and two rRNA genes. Phylogenetic results based on all PCGs and two rRNAs genes confirm *D. longibarbis* as a sister species to *D. urostriatum* in the subfamily Callichthyinae. Conclusions: In contrast to the extensive mitochondrial studies on species in the Corydoradinae, species in the Callichthyinae have been largely understudied. This study provides valuable insights into genetic diversity and evolutionary complexity by presenting the first mitochondrial genome analysis of two *Dianema* species, a genus within the Callichthyinae.

## 1. Introduction

Siluriformes, comprising 35 families, 446 genera and 2867 species, represent approximately 10.8% of all fishes and are distributed worldwide [1,2,3]. The family Callichthyidae, which includes the highly commercialized ornamental *Corydoras* catfish, is found exclusively in the Neotropics [1,4]. Species in this family possess two rows of armor-like plates and are known for their ability to breathe oxygen using the intestines as a secondary respiratory system [5]. The Callichthyidae are divided into the subfamilies Corydoradinae and Callichthyinae. Of the 200 species within Callichthyidae, only 17 belong to the Callichthyinae [6]. Molecular and phenotypic classifications strongly support the monophyly of Callichthyinae, which consists of five genera: *Callichthys*, *Dianema*, *Hoplosternum*, *Leptoplosternim*, and *Megalechis* [7,8,9].

The genus *Dianema* in Callichthyinae consists of elongated, slender catfish, comprising only two species (*Dianema longibarbis* Cope, 1872 and *Dianema urostriatum* Miranda Ribeiro, 1912) [6]. Species of the genus *Dianema* are widely distributed in the neotropical central Amazon basin and are morphologically and behaviorally distinguished from other genera in Callichthyinae [7,8]. Unlike many Corydoradinae catfishes, *Dianema* species have long barbels that help them detect food. *D. urostriatum* is easily identified by the alternating white and black stripes on its caudal fin [10], whereas *D. longibarbis* is distinguished by the absence of a black stripe on its tail.

In general, the complete mitogenome, which consists of 13 PCGs, two rRNAs, 22 tRNAs, and a control region, is currently the most reliable molecular marker in evolutionary phylogenetics of fish due to its intrinsic features such as maternal inheritance and high base substitution rate [11,12,13]. Although more than 19 complete mitogenomes have been studied in the Corydoradinae, only one species, *Hoplosternum littiorale*, which has a partial mitogenome, has been studied in the Callichthyinae [14]. Therefore, previous phylogenies of the Callichthyinae were based on morphology or partial genes of mitogenomes [7,8,9,15]. Currently, most studies of complete mitochondrial genomes in the Callichthyidae have focused on the Corydorinae, and studies on the Callichthyinae are very scarce. For the first time, this study provides information on the complete mitogenomes of the two species comprising the genus *Dianema*, *D. longibarbis* and *D. urostriatum*, which will be invaluable in resolving the cryptic molecular phylogeny of the Callichthyinae.

## 2. Materials and Methods

### 2.1. Fish and DNA Extraction

Multiple specimens of *D. longibarbis* and *D. urostriatum* were acquired through the aquarium trade (AquaPro Trading, Namyangju, Republic of Korea). Photographs of the individuals used in the experiments are provided in the Supplementary Materials (Figure S1). Muscle tissue collected from each species was prepared for total genomic DNA isolation and subsequently cataloged at the Research Institute of Basic Sciences (Incheon National University, Incheon, Republic of Korea), with Specimen ID: 2024-Callichthyidae-03 and 04. Total genomic DNA was extracted with the DNeasy Blood and Tissue Kit (Qiagen, Hilden, Germany) based on the manufacturer’s protocols. The genomic DNA extracted was assessed for purity and concentration using a Nanodrop 2000c spectrophotometer (Thermo Fisher Scientific, Waltham, MA, USA) and was then stored at −20 °C until used for analysis.

### 2.2. DNA Sequencing, Assembly, and Gene Annotation

Next-generation sequencing was executed to obtain the complete mitogenomes of *D. longibarbis* and *D. urostriatum*. This was accomplished utilizing the HiSeq platform (150 bp; HiSeq X ten; Illumina, San Diego, CA, USA) based on previously established protocols [16]. To prepare for Illumina HiSeq sequencing, a fragment library was constructed using the TruSeq DNA Sample Preparation Kit (Illumina, San Diego, CA, USA) in accordance with the manufacturer’s suggestions at Macrogen, Inc. (Seoul, Republic of Korea). This entailed the random fragmentation of isolated total genomic DNA of each fish, followed by the ligation process with 5′ and 3′ adapters. Subsequently, the libraries underwent sequencing on the Illumina HiSeq platform, and the synthesized paired-end raw reads were tested through a stringent quality control procedure using FastQC version 0.11.9 [17]. After the demultiplexing process, only index pairs that matched were retrieved for further procedures. The raw read data underwent a meticulous quality trimming test, during which sequences for the 5′ and 3′ adapters, reads including low-quality sequences, reads with more than 10% unknown sequences, and reads containing ambiguous sequences were diligently removed using Trimmomatic [18], ultimately yielding a high-quality assembly. From an initial total of 4,560,049,755 and 4,800,833,497 raw reads for *D. longibarbis* and *D. urostriatum*, respectively, we obtained a final 30,259,360 and 31,853,978 filtered reads. To obtain circular contigs of the *D. longibarbis* and *D. urostriatum* mitogenomes, de novo assemblies were performed using different k-mers via NOVOplasty [19]. The average depth of coverage is provided in Figure S2. The resultant consensus sequence of the contig was annotated using MITOS2 [20]. The creation of mitochondrial genome maps for *D. longibarbis* and *D. urostriatum* were conducted through Proksee [21].

### 2.3. Mitogenome Analysis

The nucleotide composition and codon frequency and relative synonymous codon usage (RSCU) of both mitogenomes were calculated through MEGA 11 version 11.0.13 [22]. AT-skew and GC-skew for each mitogenome were analyzed with the equations of AT-skew = (A − T)/(A + T) and GC-skew = (G − C)/(G + C) for each fish, respectively. Prediction on the secondary structure for each tRNA was performed in the tRNASCAN webserver [23]. The control region sequences of 24 species belonging to Callichthyidae were obtained in the NCBI database. The tandem repeat sequences of the control region were predicted for each species using tandem repeat finder [24]. Nucleotide diversity (π) and nucleotide distances via the K2P model [25] for 13 PCGs of the 24 species of Callichthyidae in Table S1 were calculated using MEGA 11 version 11.0.13 [22]. The Ka/Ks values were analyzed with DnaSP version 6.12.03 [26].

### 2.4. Phylogenetic Analysis

To assess the phylogenetic relationships of *D. longibarbis* and *D. urostriatum*, maximum-likelihood (ML) and Bayesian inferences (BI) phylogenetic trees were constructed using concatenated amino acid coding sequence from all PCGs and two rRNA genes from the mitogenomes of *D. longibarbis* and *D. urostriatum*, with 34 mitogenomes retrieved from Siluriformes (24 species in Callichthyidae, seven species in Loricariidae, two species in Siluridae, and one species in Trichomycteridae,). The references for the GenBank accession number of each fish used in the phylogenetic analysis are appended in Supplementary Materials (Table S1). The 13 PCGs and two rRNA genes of each fish were obtained from the NCBI database. Sequences of the 13 PCGs and two rRNA genes were aligned through the L-INS-I algorithm with MAFFT version 7.490 [27]. Redundant gaps in each gene were trimmed using trimAl version 1.4 [28]. The modified sequences were then used to generate two matrices, one for the 13 PCGs and another for the combination of two rRNA + 13 PCGs, using SequenceMatrix version 1.8.1 and subsequently underwent conversion process to obtain nexus format [29]. To find the best substitution model for the sequences, the ModelFinder in IQ-TREE2 version 2.0.7 was used (Table S2) [30]. The ML-based phylogenetic result was established with 1000 replications of ultrafast bootstrapping with the optimal substitution model based on Bayesian Information Criterion (BIC) in IQ-TREE2 version 2.0.7 [31]. The BI-based phylogenetic tree was constructed using MrBayes program version 3.2.7, based on the best substitution model estimated through Akaike Information Criterion (AIC) [32]. Two independent Markov Chain Monte Carlo (MCMC) samplings of one million generations were applied using four chains and the tree was sampled every 100 generations. The effective sample size (ESS) was calculated with Tracer version 1.7.2, and all metrics were above 200, except for 25% burn-in [33]. Of the generated trees, the first 25% were removed as burn-in using LogCombiner version 2.7, and a consensus phylogenetic tree was constructed with TreeAnnotator version 2.7.6 [34]. The generated ML and BI trees were edited and visualized using Figtree version 1.4.4 [35].

## 3. Results

### 3.1. Mitogenome Structure and Base Composition

**Figure 1:**
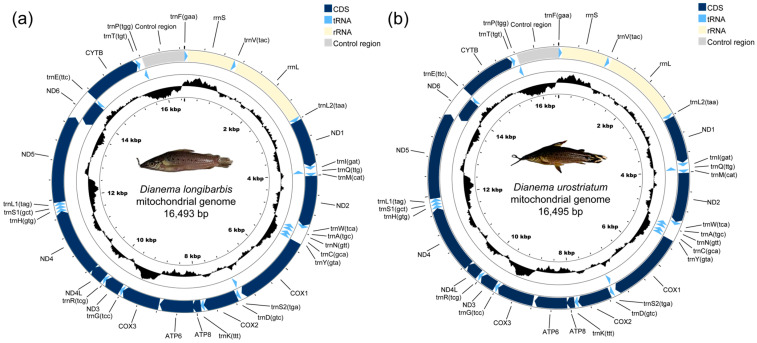
The circular maps of the assembled mitogenomes of (**a**) *D. longibarbis* and (**b**) *D. urostriatum* consist of 13 PCGs, 22 tRNA genes, and two rRNA genes. Genes encoded on the reverse strand are highlighted inside the circle, while those on the forward strand are marked outside the circle.

The complete mitogenomes of *D. longibarbis* and *D. urostriatum* are 16,493 bp and 16,495 bp long, respectively (GenBank accession no. PP737535 and PP790961) (Figure 1a,b and Table 1). In comparison to the mitogenomes of the other 20 species in the Callichthyidae, which range from 16,531 to 16,916 bp, these are among the shortest. Both mitogenomes contain a total of 13 PCGs, two rRNA genes (*rrnS* (12S) and *rrnL* (16S)), 22 tRNA genes, and one control region (Figure 1a,b and Table 1).

The overall base compositions of the complete mitogenomes of *D. longibarbis* and *D. urostriatum* are provided in Table 2. The AT content of the mitogenome is 59.32% in *D. longibarbis* and 58.73% in *D. urostriatum*, with respective values of 59.56% and 58.84% for PCGs, 56.74% and 57.03% for tRNAs, 57.01% and 56.61% for rRNAs, and 66.51% and 64.70% for the control region (Table 2).

There were 13 intergenic sequences between adjacent genes within the mitogenomes of the two species, ranging in length from 1 to 30 bp, and seven overlap sequences, ranging in length from 1 to 13 bp (Table 1). The intergenic sequence between the *atp6* and *cox3* genes is a synapomorphy feature to Callichthyidae, with a length of 29 bp (TATTTAAATCTAGCTCTATTAAATTAATT) in *D. longibarbis* and 30 bp (TATCTAAAACTATACTAAACTAAATAATTA) in *D. urostriatum* (Table 1).

### 3.2. Protein-Coding Genes

In both *D. longibarbis* and *D. urostriatum*, the *nad6* gene is located on the light (L) strand, and the 12 PCGs (*atp6*, *atp8*, *cox1*, *cox2*, *cox3*, *nad1*, *nad2*, *nad3*, *nad4*, *nad4l*, *nad5*, and *cytb*) are located on the heavy (H) strand (Table 1). All 13 PCGs initiate with the conventional start codon ATG, except for the *cox1* gene, which begins with GTG as its start codon. Six PCGs (*nad1*, *nad2*, *atp6*, *atp8*, *nad4l*, *nad5*, and *nad6*) terminate with complete stop codons (TAA or TAG), while the cox1 gene terminates with AGG (Table 1). In addition, *cox2*, *nad4*, and *cytb* terminate with an incomplete stop codon (T-), and *cox3* terminates with an incomplete stop codon (TA-).

**Figure 2:**
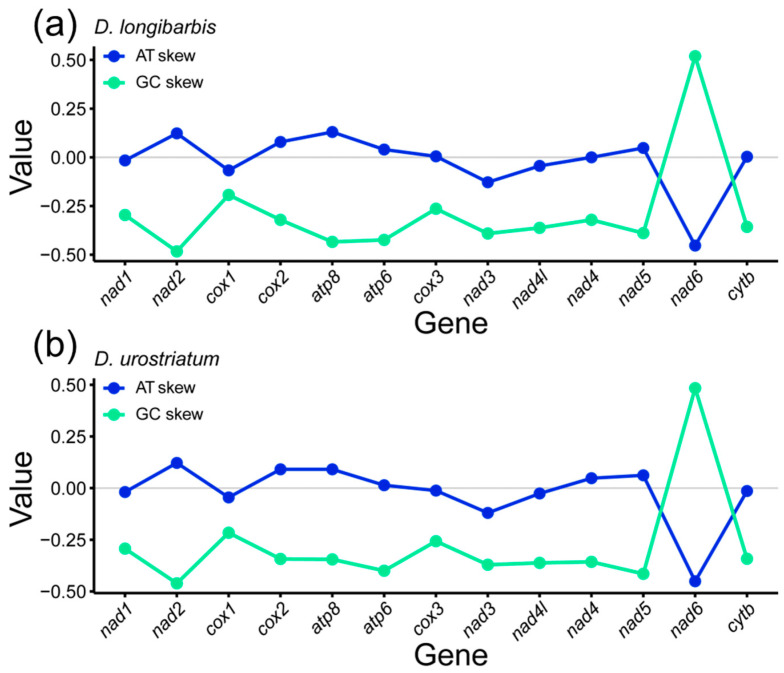
AT and GC skewness of 13 PCGs in the mitogenomes of (**a**) *D. longibarbis* and (**b**) *D. urostriatum*.

The AT skew of the 13 PCGs for *D. longibarbis* was zero or positive for *nad2* (0.123), *cox2* (0.079), *atp8* (0.130), *atp6* (0.040), *cox3* (0.005), *nad4* (0.000), and *nad5* (0.048). For *D. urostriatum*, the AT skew was positive for *nad2* (0.122), *cox2* (0.091), *atp8* (0.091), *atp6* (0.014), *nad4* (0.048), and *nad5* (0.062), and negative for the other genes (Figure 2a,b). On the other hand, the GC skew was negative for both species, except for the *nad6* gene (Figure 2a,b).

**Figure 3:**
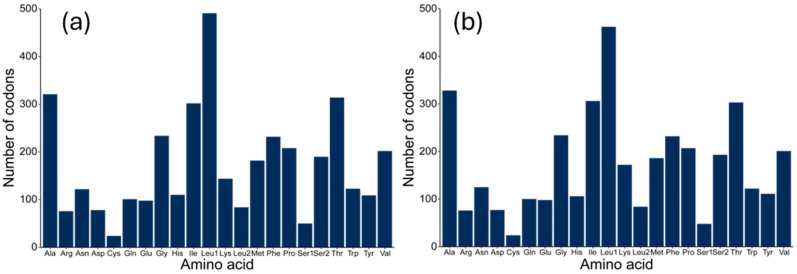
Frequency of amino acids in the 13 PCGs of the (**a**) *D. longibarbis* and (**b**) *D. urostriatum* mitogenomes.

Excluding the termination codon, the total number of codons in the 13 PCGs of both species was the same at 3795. For *D. longibarbis*, Leucine (Leu, 634), Alanine (Ala, 328), Isoleucine (Ile, 306), and Threonine (Thr, 303) were the most frequent, followed by Cysteine (Cys, 24), Arginine (Arg, 76), Aspartic Acid (Asp, 77), and Lysine (Lys, 84), which were the least frequent (Figure 3a and Table S3). In the case of *D. urostriatum*, Leucine (Leu, 635), Alanine (Ala, 321), Threonine (Thr, 314), and Isoleucine (Ile, 302) were the most frequent, and Cysteine (Cys, 24) Arginine (Arg, 76) Aspartic Acid (Asp, 78), and Lysine (Lys, 84) were the least frequent (Figure 3b and Table S4). The codon frequencies in the two species were similar, but the third most common codon was Ile in *D. longibarbis* and Thr in *D. urostriatum*.

**Figure 4:**
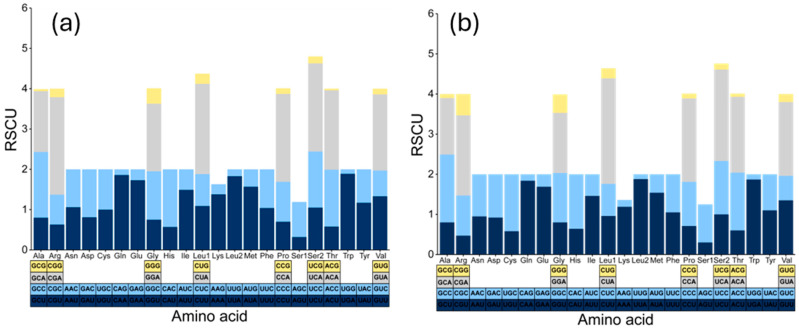
Relative synonymous codon usage (RSCU) in the 13 PCGs of the (**a**) *D. longibarbis* and (**b**) *D. urostriatum* mitogenomes.

Regarding the RSCU results for *D. longibarbis* and *D. urostriatum* (Figure 4a,b and Tables S3 and S4), in *D. longibarbis*, CGA (Arg, 2.42), CUA (Leu1, 2.24), UCA (Ser2, 2.19), and CCA (Pro, 2.18) had the highest RSCU values, while in *D. urostriatum*, CUA (Leu1, 2.63), UCA (Ser2, 2.28), CCA (Pro, 2.08), and CGA (Arg, 2.00) had the highest RSCU values. On the other hand, ACG (Thr, 0.04), GCG (Ala, 0.05), UGG (Trp, 0.11), and CAG (Gln, 0.14) had the lowest RSCU values in *D. longibarbis*, followed by ACG (Thr, 0.08), GCG (Ala, 0.10), AAG (Lys, 0.12), and CCG (Pro, 0.12) in *D. urostriatum* (Figure 4a,b and Tables S3 and S4). In both species, a strong anti-bias G was analyzed at the third position of codons.

**Figure 5:**
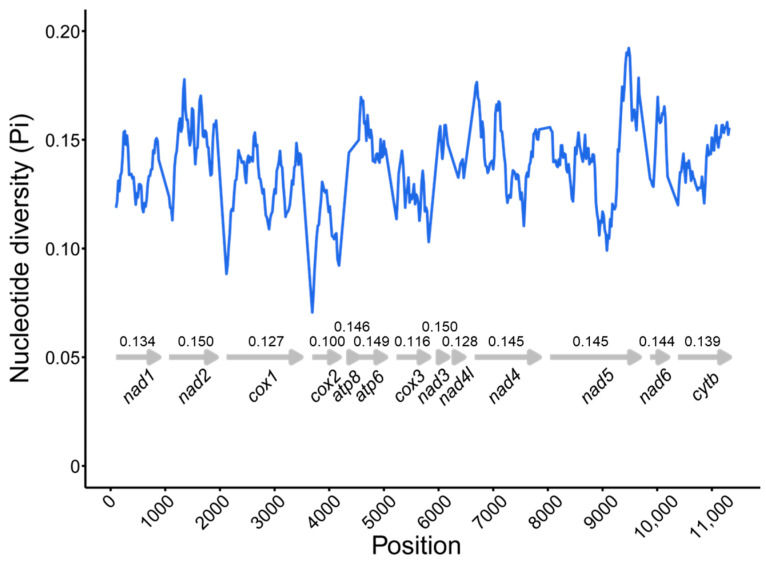
The nucleotide diversity by position of concatenated 13 PCGs for 24 species in the Callichthyidae. The arrows indicate the position of each PCG, and the numbers indicate the pi value of that PCG. Detailed information about the species is provided in Table S1.

**Figure 6:**
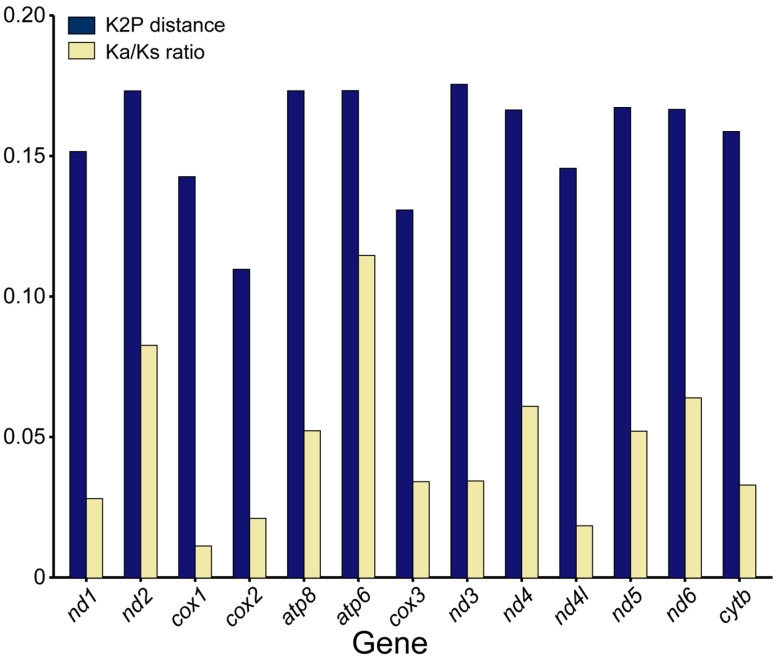
K2P distance and Ka/Ks ratio for 13 PCGs belonging to 24 species of the Callichthyidae. Detailed information about the species is provided in Table S1.

To identify hidden evolutionary information about *D. longibarbis* and *D. urostriatum*, genetic distance, nucleotide diversity, and Ka/Ks ratios were analyzed for their 13 PCGs among species belonging to the Callichthyidae (Figure 5 and Figure 6). In the nucleotide diversity (Pi) observed in this study, *cox2* (0.100), *cox3* (0.116), and *cox1* (0.127) showed the lowest values, while *nad3* (0.150), *nad2* (0.150), and *atp6* (0.149) showed the highest values (Figure 5). For K2P distance, *cox2* (0.110), *cox3* (0.131), and *cox1* (0.143) had the lowest values, followed by *nad3* (0.175), *atp6* (0.173), and *atp8* (0.173) (Figure 6). As a common result of Pi and K2P distance, the *cox* genes were found to have the highest conservation as it exhibited low genetic diversity and low intergenic distance. In addition, the genes with the lowest Ka/Ks ratio were *cox1* (0.011), *nad4l* (0.018), and *cox2* (0.021), and the genes with the highest Ka/Ks ratio were *atp6* (0.115), *nad2* (0.083), and *nad6* (0.064) (Figure 6).

### 3.3. tRNAs, rRNAs and Control Region

In both species, the lengths of 22 tRNAs ranged from 67 to 75 bp. Fourteen tRNAs (*trnF*, *trnV*, *trnI*, trnM, *trnN*, *trnA*, *trnY*, *trnD*, *trnK*, *trnH*, *trnS1*, *trnL1*, *trnE*, and *trnT*) were located on the H strand, while the remaining eight tRNAs (*trnL2*, *trnQ*, *trnW*, *trnC*, *trnS2*, *trnG*, *trnR*, and *trnP*) were located on the L strand (Table 1). The predicted secondary structure of tRNAs had a cloverleaf structure except for *trnS1*, which does not have dihydrouridine (DHU) arm structure (Figures S3 and S4).

For *D. longibarbis*, the *rrnS* was 949 bp long and the *rrnL* was 1641 bp, while for *D. urostriatum*, the *rrnS* was 950 bp and the *rrnL* was 1642 bp (Table 1). All rRNAs were located on the H strand.

Interestingly, the control regions of *D. longibarbis* and *D. urostriatum* were 869 and 864 bp in length, respectively, which is the smallest length observed, considering that the control regions of other Callichthyidae species range from 904 to 1299 bp in length (Figure S5).

### 3.4. Phylogenetic Analysis

**Figure 7:**
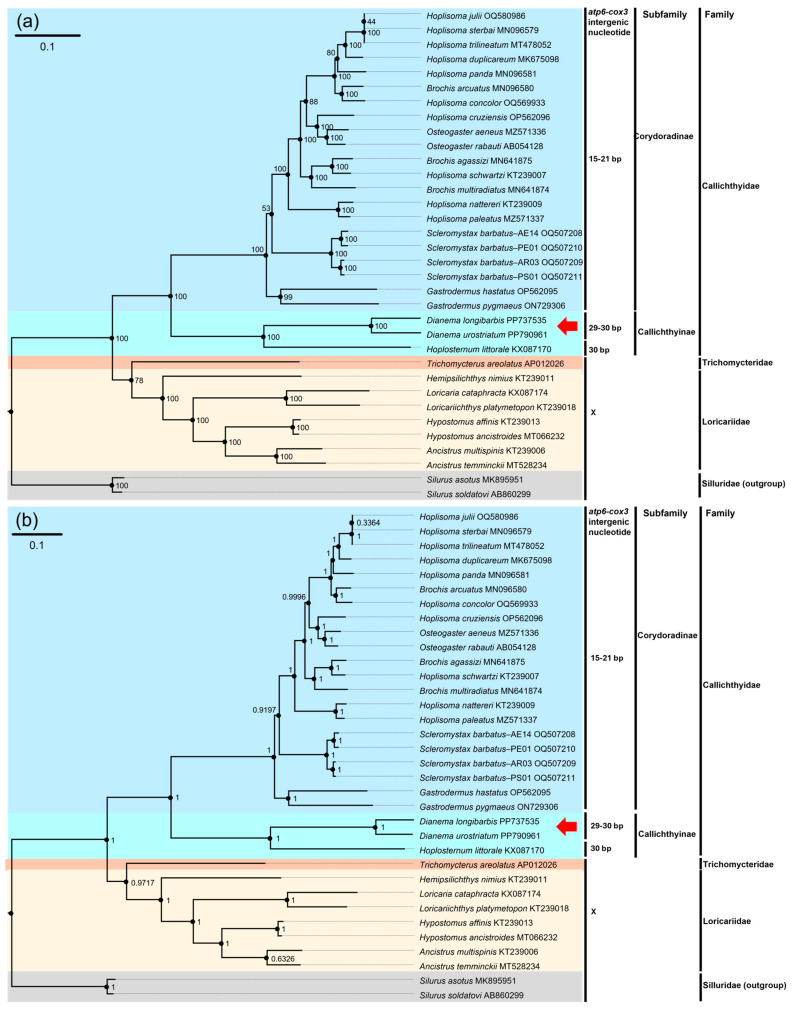
The phylogenetic trees of 34 published Siluriformes mitogenomes, including that of *D. longibarbis* and *D. urostriatum*, constructed based on the concatenated nucleotide sequences of 13 PCGs and two rRNAs. (**a**) The numbers on the nodes indicate ML bootstrap percentages. (**b**) The numbers on the nodes indicate Bayesian posterior probability. GenBank accession number for the published sequence of each species is appended. The base pair numbers indicated the number of intergenic nucleotides between two genes, *atp6* and *cox3*. The red arrow represents the two catfish species analyzed in this study. References for the mitogenome data used in this analysis are appended in Table S1.

The phylogenetic results were constructed using either Bayesian interference or the maximum likelihood estimate model, with 13 PCGs and 13 PCGs + 2rRNAs, incorporating species from three families within the suborder Loricarioidei (Callichthyidae, Loricariidae, and Trichomycteridae), and species from the family Siluridae within the suborder Siluroidei as outgroups (Figure 7, Figures S6 and S7). The phylogenetic tree using 13 PCGs and the tree using 13 PCGs + 2rRNA shared the same topology). Both the ML and BI trees constructed monophyletic clades for Callichthyidae, Trichomycteridae, Loricariidae, and Silluridae. Callichthyinae and Corydoradinae were separated within Callichthyidae with high posterior probability (BI = 1.0) and bootstrap percentage (ML = 100), forming a single clade. *D. longibarbis* and *D. urostriatum* were sister species to each other, clustered in the same clade with *Hoplosternum littorale* within Callichthyinae and had high support values (ML = 100 and BI = 1.0).

## 4. Discussion

The mitogenomes of *D. longibarbis* and *D. urostriatum*, two species belonging to the Dianema observed in this study, were 16,493 and 16,495 bp in length, respectively, and consisted of 13 PCGs, two rRNAs, 22 tRNAs, and one control region. Their structure and arrangement were identical to those of common vertebrate mitogenomes [12]. In 13 PCGs, *D. longibarbis* and *D. urostriatum* contain the same initiation and termination codons, and additionally, the incomplete termination codons observed in *cox2*, *cox3*, *nad4*, and *cytb* are commonly observed in PCGs of teleost mitogenomes, and these incomplete termination codons are considered to function normally during post-transcriptional polyadenylation [36,37].

Insertion sequences of 29 bp (TATTTAAATCTAGCTCTATTAAATTAATT) and 30 bp (TATCTAAAACTATACTAAACTAAATAATTA) were observed in *D. longibarbis* and *D. urostriatum*, respectively. A similarly long intergenic sequence of 30 bp (CACATAATTAATCACATAAATTAAATCT) was also observed in *H. littiorale*, another species in the Callichthyinae, while a shorter intergenic sequence of 15–21 bp was observed in Corydoradinae [14,38,39,40,41,42,43,44,45,46,47,48]. Given the high interspecific similarity in Corydoradinae, it was suggested that the *atp6*-*cox3* intergenic sequence could function as a molecular marker for species classification, whereas in Callichthyinae, no intergenic sequence similarity was observed, except for length [48]. The presence of long intergenic sequences in Callichthyinae could be due to sequence duplication, as previously hypothesized, but the lack of sequence identity between the genomes of *Dianema* and *Hoplosternum* suggests that duplication may not be the underlying cause [8]. Nevertheless, the reason for the longer intergenic sequences observed in Callichthyinae remains unclear, and it is also unknown where these intergenic sequences originated. Further studies of the mitogenomes of Callichthyinae species are needed to address the mystery of the long intergenic sequences in this subfamily.

Ka/Ks ratios below 1 were observed for all PCGs, indicating that the genes are under purifying selection [49]. Although in the previous comparison of PCGs belonging to the Corydoradinae, *atp8* showed higher Pi and K2P distance values than *cox1*, the results of the analysis of PCGs in the Callichthyinae indicate that the *atp8* gene in the *Hoplosternum* or *Dianema* genera is under high evolutionary pressure [38,45,48].

Deletion of the DHU arm in *trnS1* is a common feature observed in a variety of teleosts, as well as previous studies of the Callichthyidae mitogenomes [36,38]. Despite being a non-coding region, the control region is known to contain promoter regions and is where the origin of replication is located [50]. Although all species in the Callichthyidae, except *D. longibarbis* and *D. urostriatum*, were found to have tandem repeat sequences in the upstream position of the control region, the absence of tandem repeats in the control region of these two species may account for the unusually short length of the control region in *D. longibarbis* and *D. urostriatum*.

Discordance among species in the genera of *Brochis*, *Hoplisoma*, and *Osteogaster* within the Corydoradinae was consistent with previous studies [38,45,48]. To date, studies of the evolutionary position of the Callichthyinae have not shown consistent results. For example, the cladogram based on osteological features recovered the topology of ((((*Hoplosternum* + *Dianema*) + *Megalechis*) + *Leptophosternum*) + *Callichthys*) [7], and the phylogenetic trees based on mitochondrial genes (two rRNAs, two tRNAs, and *nad4*) and mitochondrial genes (two rRNAs, two tRNAs, and *nad4*) + the nuclear gene (*sia*) followed the topology of ((((*Megalechis* + *Leptophosternum*) + *Callichthys*) + *Hoplosternum*) + *Dianema*) [8,9]. In addition, a cladogram constructed from morphological features and mitochondrial genes (two rRNAs, *nad4*, and *cytb*) + the nuclear gene (*rag1*) represented the topology of (((*Hoplosternum* + *Dianema*) + (*Callichthys* + (*Megalechis* + *Leptophosternum*)))) [15]. Phylogenetic classifications constructed using partial mitogenomes and partial mitogenomes + nuclear genes commonly indicate that *Dianema* is the primitive species of Callichthyinae. However, this study suggests that *H. littorale* is a primitive species within Callichthyinae. This discrepancy may result from the fact that only *D. longibarbis*, *D. urostriatum*, and *H. littorale* were included in this study, and further studies of the mitogenomes of Callichthyinae species are needed to resolve this issue.

## 5. Conclusions

This study describes the complete mitogenomes of *D. longibarbis* and *D. urostriatum*, the only species in the genus *Dianema*. The characteristic short length of the control region in *Dianema* is attributed to the absence of tandem repeats, which are common in other Callichthyidae species. Furthermore, the increased nucleotide diversity and divergence of the *atp8* gene, compared to species in the Corydoradinae, suggest that the *atp8* gene in the Callichthyinae is under strong evolutionary pressure. The constructed phylogenetic tree indicated that Callichthyinae and *Dianema* were supported as monophyletic groups based on strong support from both Bayesian inference (BI) and maximum likelihood (ML) analyses. Nevertheless, unlike previous studies, whether *Dianema* represents a primitive genus in the Callichthyidae requires further investigation.

## Figures and Tables

**Table 1:** Detailed information on the *D. longibarbis* and *D. urostraitum* mitogenomes including the start and end positions of each gene, the number of overlapping and *atp6*-*cox3* intergenic nucleotides, the strand positions of genes, as well as the start and stop codons of entire PCGs and the anticodons of tRNAs.

Gene	Position				Codon			
	*D. longibarbis* */D. urostriatum*				Strand	Start	Stop	Anticodon
	Start	End	Length (bp)	Intergenic Nucleotide				
*trnF*	1/1	68/68	68/68	0/0	H			GAA
*rrnS*	69/69	1017/1018	949/950	0/0	H			
*trnV*	1018/1019	1089/1090	72/72	0/0	H			TAC
*rrnL*	1111/1113	2751/2754	1641/1642	0/0	H			
*trnL2*	2752/2755	2826/2829	75/75	0/0	H			TAA
*nad1*	2827/2830	3798/3801	972/972	8/8	H	ATG	TAG	
*trnI*	3807/3810	3878/3881	72/72	−2/−2	H			GAT
*trnQ*	3877/3950	3947/3880	71/71	−1/−1	L			TTG
*trnM*	3947/3950	4016/4019	70/70	0/0	H			CAT
*nad2*	4017/4020	5063/5064	1047/1045	−2/−2	H	ATG	TAG	
*trnW*	5062/5065	5133/5135	72/71	2/2	H			TCA
*trnA*	5136/5206	5204/5138	69/69	1/1	L			TGC
*trnN*	5206/5280	5278/5208	73/73	30/30	L			GTT
*trnC*	5309/5378	5375/5311	67/68	−1/−2	L			GCA
*trnY*	5375/5447	5444/5378	70/70	1/1	L			GTA
*cox1*	5446/5449	7008/7011	1563/1563	−13/−13	H	GTG	AGG	
*trnS2*	6996/7069	7066/6999	71/71	4/4	L			TGA
*trnD*	7071/7074	7140/7143	70/70	3/6	H			GTC
*cox2*	7144/7150	7834/7840	691/691	0/0	H	ATG	T-	
*trnK*	7835/7841	7908/7914	74/74	1/1	H			TTT
*atp8*	7910/7916	8077/8083	168/168	10/10	H	ATG	TAG	
*atp6*	8068/8074	8751/8757	684/684	29/30	H	ATG	TAA	
*cox3*	8781/8788	9565/9572	785/785	0/0	H	ATG	TA-	
*trnG*	9565/9572	9635/9643	71/72	0/0	H			TCC
*nad3*	9636/9644	9986/9994	351/351	−2/−2	H	ATG	TAG	
*trnR*	9985/9993	10,054/10,062	70/70	0/0	H			TCG
*nad4l*	10,055/10,063	10,351/10,359	297/297	7/7	H	ATG	TAA	
*nad4*	10,345/10,353	11,725/11,733	1381/1381	0/0	H	ATG	T-	
*trnH*	11,726/11,734	11,795/11,803	70/70	0/0	H			GTG
*trnS1*	11,796/11,804	11,862/11,870	67/67	0/0	H			GCT
*trnL1*	11,863/11,871	11,935/11,943	73/73	0/0	H			TAG
*nad5*	11,936/11,944	13,759/13,767	1824/1824	4/4	H	ATG	TAA	
*nad6*	13,756/14,279	14,271/13,764	516/516	0/0	L	ATG	TAA	
*trnE*	14,272/14,348	14,340/14,280	69/69	6/5	L			TTC
*cytb*	14,347/14,354	15,484/15,491	1138/1138	0/0	H	ATG	T-	
*trnT*	15,485/15,492	15,556/15,563	72/72	−2/−2	H			TGT
*trnP*	15,555/15,631	15,624/15,562	70/70	0/0	L			TGG
*C.R.*	15,625/15,632	16,493/16,495	869/864	0/0				

**Table 2:** Detailed information on the nucleotide composition of the whole mitogenome (Total) and its PCGs, tRNAs, rRNAs, and control region (C.R.) in *D. longibarbis* and *D. urostriatum*.

Gene	Nucleotide Composition						
	*D. longibarbis* */D. urostriatum*						
	Size (bp)	A (%)	T (%)	C (%)	G (%)	A + T (%)	G + C (%)
Total	16,493/16,495	31.79/31.69	27.53/27.04	25.86/26.36	14.82/14.91	59.32/58.73	40.68/41.27
*PCGs*	11,413/11,422	29.64/29.50	29.92/29.34	26.20/26.81	14.24/14.35	59.56/58.84	40.44/41.16
*tRNAs*	1556/1556	28.98/29.13	27.76/27.90	20.24/19.95	23.01/23.02	56.74/57.03	43.25/42.97
*rRNAs*	2611/2592	34.92/34.49	22.09/21.77	22.90/23.45	20.09/23.45	57.01/56.61	42.99/43.39
*C.R.*	869/864	34.75/34.49	31.76/30.21	18.87/19.91	14.61/15.39	66.51/64.70	33.49/35.30

## Data Availability

The Bioproject, Biosample, and SRA accession numbers for *D. longibarbis* are PRJNA1229777, SAMN47142742, and SRR32520581, respectively, while those for *D. urostriatum* are PRJNA1229778, SAMN47142749, and SRR32520608, respectively. The data supporting these findings are publicly available at the National Center for Biotechnology Information under the accession numbers PP737535 (*D. longibarbis*) and PP790961 (*D. urostriatum*).

## Supplementary Materials

The following supporting information can be downloaded at: https://www.mdpi.com/article/10.3390/genes16030355/s1, Figure S1: Photographs of (a) *D. longibarbis* and (b) *D. urostriatum* used for DNA extraction; Figure S2: The coverage depth for each genomic position in (a) *D. longbibarbis* and (b) *D. urostriatum*; Figure S3: The predicted secondary structure of tRNAs in the *D. longibarbis* mitogenome; Figure S4: The predicted secondary structure of tRNAs in the *D. urostriatum* mitogenome; Figure S5: Tandem repeat sequences in the control regions of 24 species in the Callichthyidae; Figure S6: A maximum likelihood (ML) phylogenetic tree of 34 published mitogenomes of Siluriformes, including mitogenomes of *D. longibarbis* and *D. urostriatum*, based on the concatenated nucleotide sequences of 13 PCGs; Figure S7: A Bayesian phylogenetic tree of 32 published mitogenomes of Siluriformes, including mitogenomes of *D. longibarbis* and *D. urostriatum*, based on the concatenated nucleotide sequences of 13 PCGs; Table S1: The mitogenome data used in phylogenetic analysis; Table S2: Best substitution model used for phylogenetic trees; Table S3: Details on codon count and RSCU in the *D. longibarbis* mitogenome; Table S4: Details on codon count and RSCU in the *D. urostriatum* mitogenome. References [14,38,40,41,42,43,44,45,46,47,48,51,52,53,54,55,56,57,58,59] are cited in the Supplementary Materials.
