# Supplementary material for: The First Complete Mitochondrial Genomes for the Genus Dianema (Siluriformes: Callichthyidae): Dianema longibarbis and D. urostriatum
Source: Genes (Basel). 2025 Mar 20;16(3):355. doi: 10.3390/genes16030355 (PMC11941792; doi:10.3390/genes16030355)
Supplement: Supplementary file 1 [file genes-16-00355-s001.zip › genes-3533085-supplementary.pdf]

< Supplementary Information >

The first complete mitochondrial genomes for the genus *Dianema* (Siluriformes: Callichthyidae): *Dianema longibarbis* and *D. urostriatum*

Seong Duk Do[a], Jae-Sung Rhee[a,b,c,*]

[a]Department of Marine Science, College of Natural Sciences, Incheon National University, Incheon, South Korea; [b]Research Institute of Basic Sciences, Incheon National University, Incheon, South Korea; [c]Yellow Sea Research Institute, Incheon, South Korea

*Correspondence:
jsrhee@inu.ac.kr (J.-S. Rhee)

TABLE OF CONTENTS

SUPPLEMENTARY INFORMATION..3
Figure S1. 3
Figure S2. 4
Figure S3. 5
Figure S4. 6
Figure S5. 7
Figure S6 8
Figure S7. 10
Table S1...10
Table S2...11
Table S3...12
Table S4...13

SUPPLEMENTARY INFORMATION

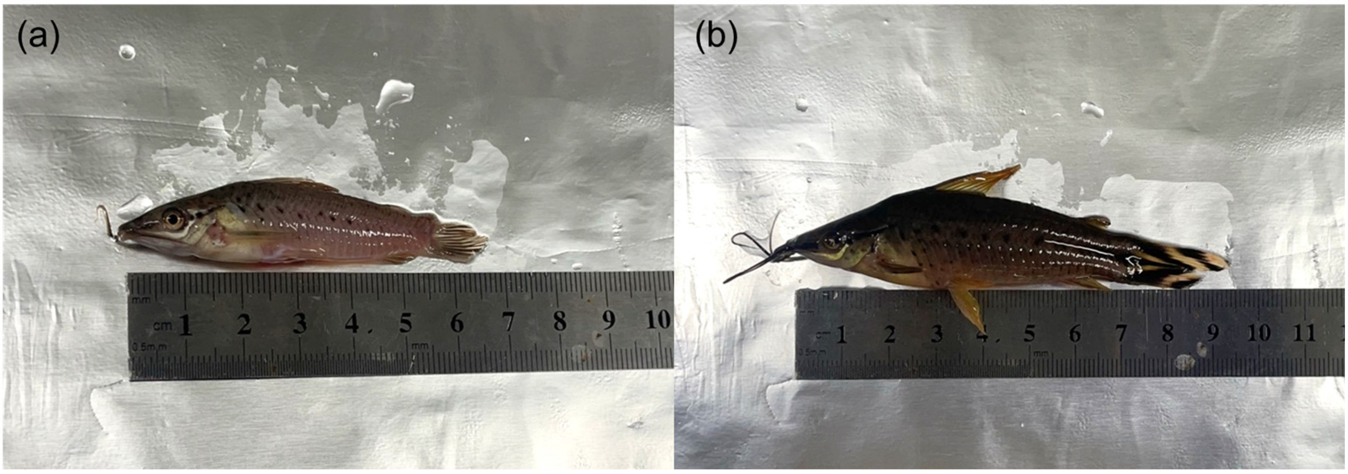

Figure S1. Photographs of (a) *D. longibarbis* and (b) *D. urostriatum* used for DNA extraction. The photographs were taken by the authors.

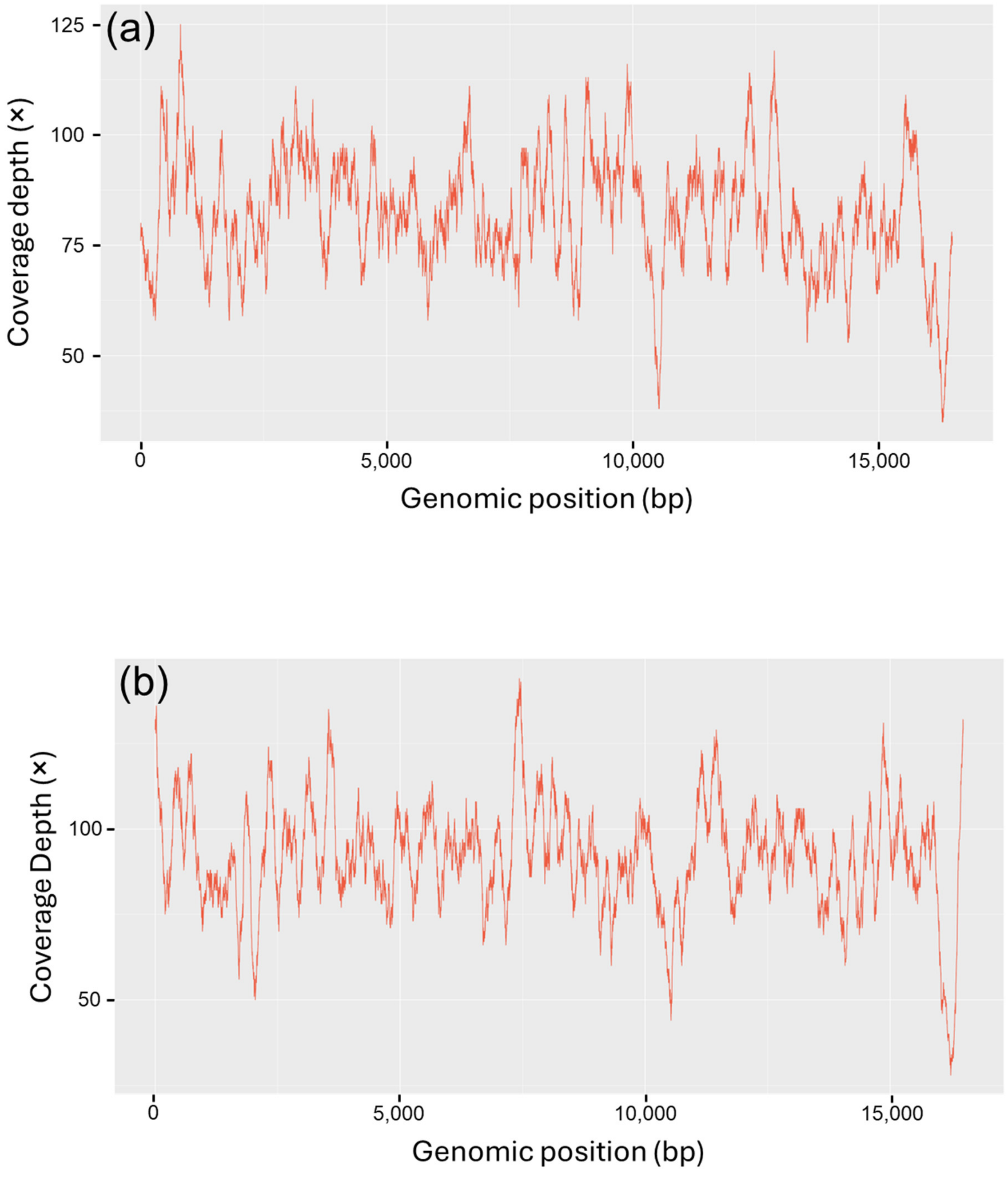

Figure S2. The coverage depth for each genomic position in (a) *D. longbibarbis* and (b) *D. urostriatum*.

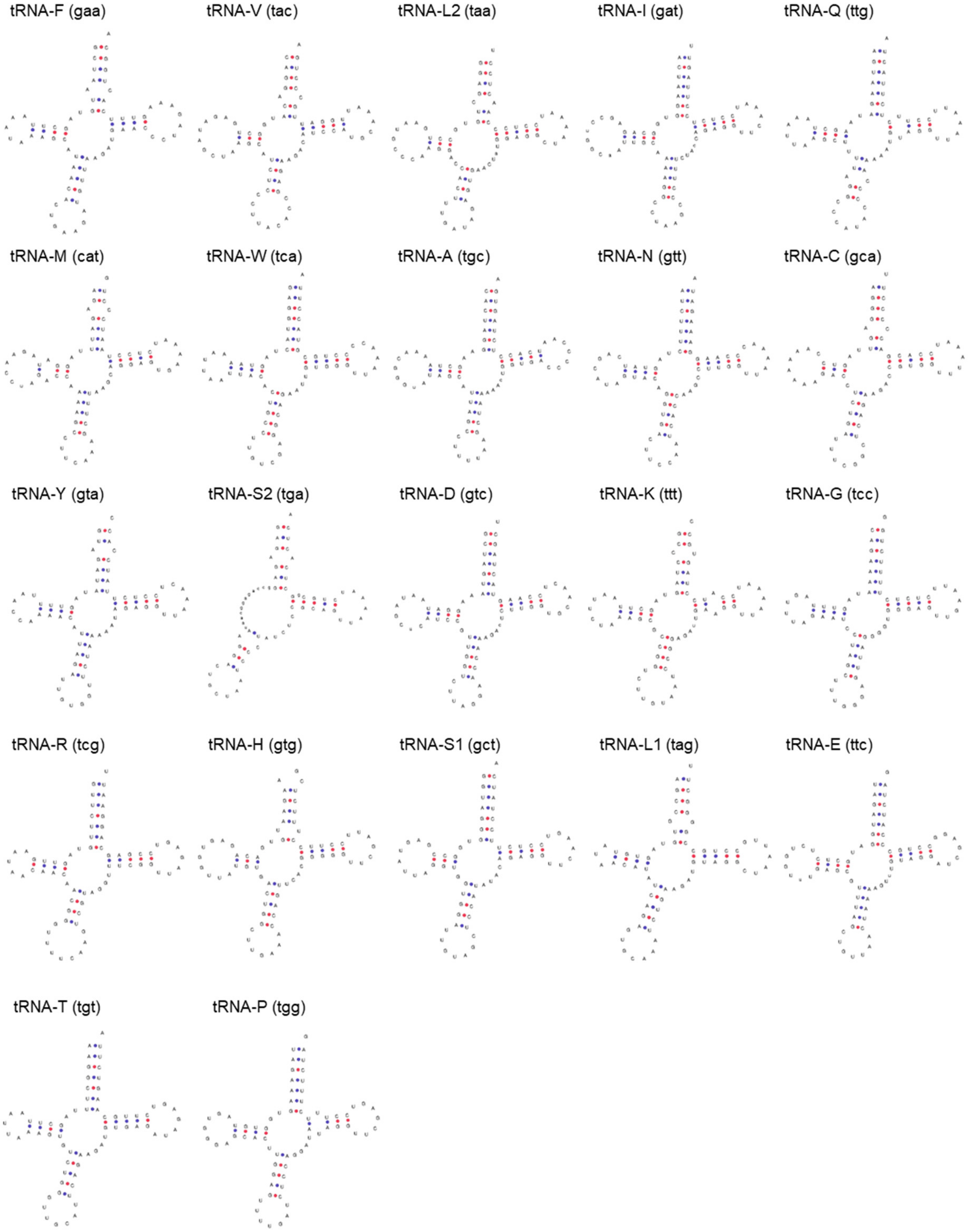

Figure S3. The predicted secondary structure of tRNAs in the *D. longibarbis* mitogenome including information on the anticodon sequence of each tRNA and the corresponding amino acids they transport.

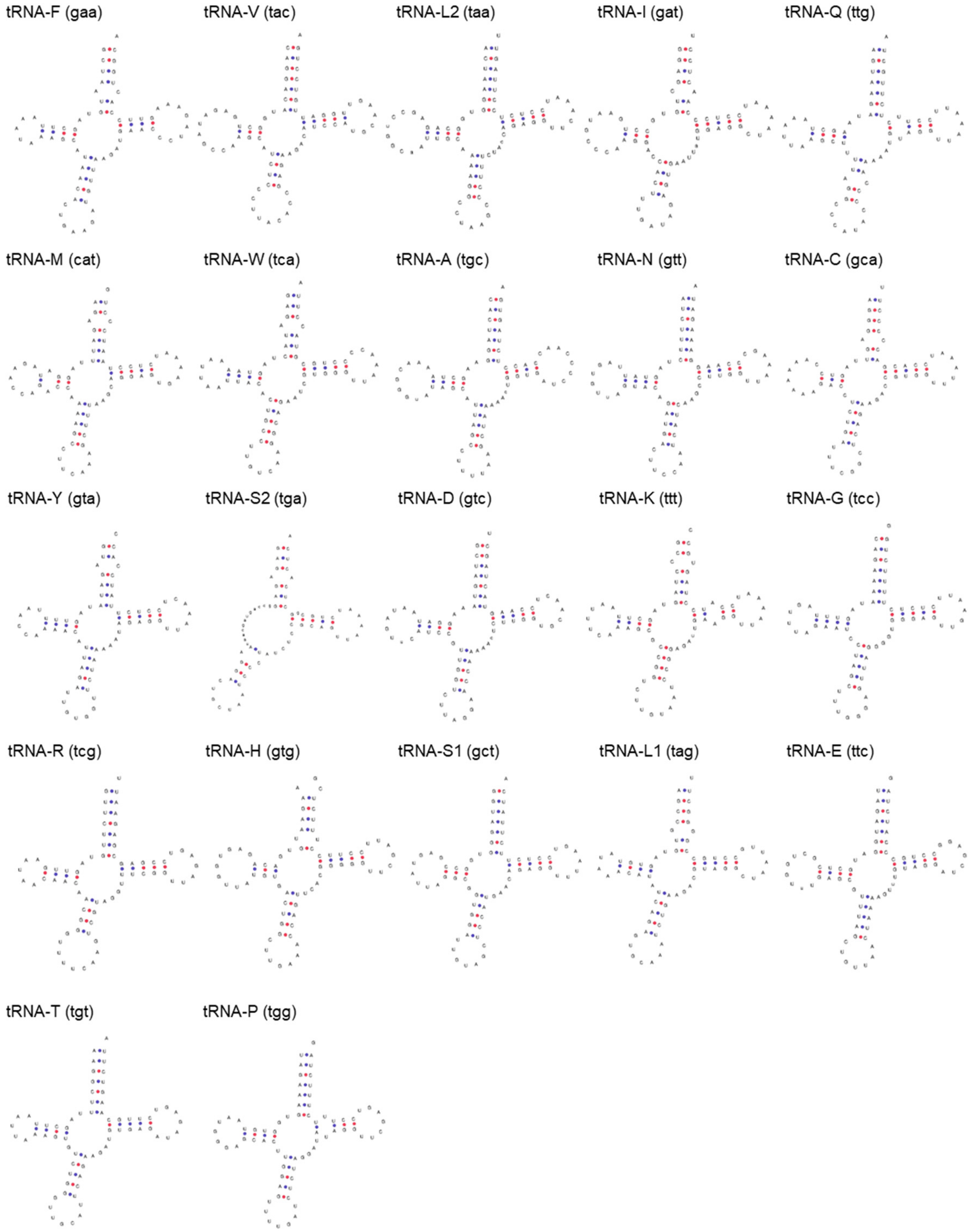

Figure S4. The predicted secondary structure of tRNAs in the *D. urostriatum* mitogenome including information on the anticodon sequence of each tRNA and the corresponding amino acids they transport.

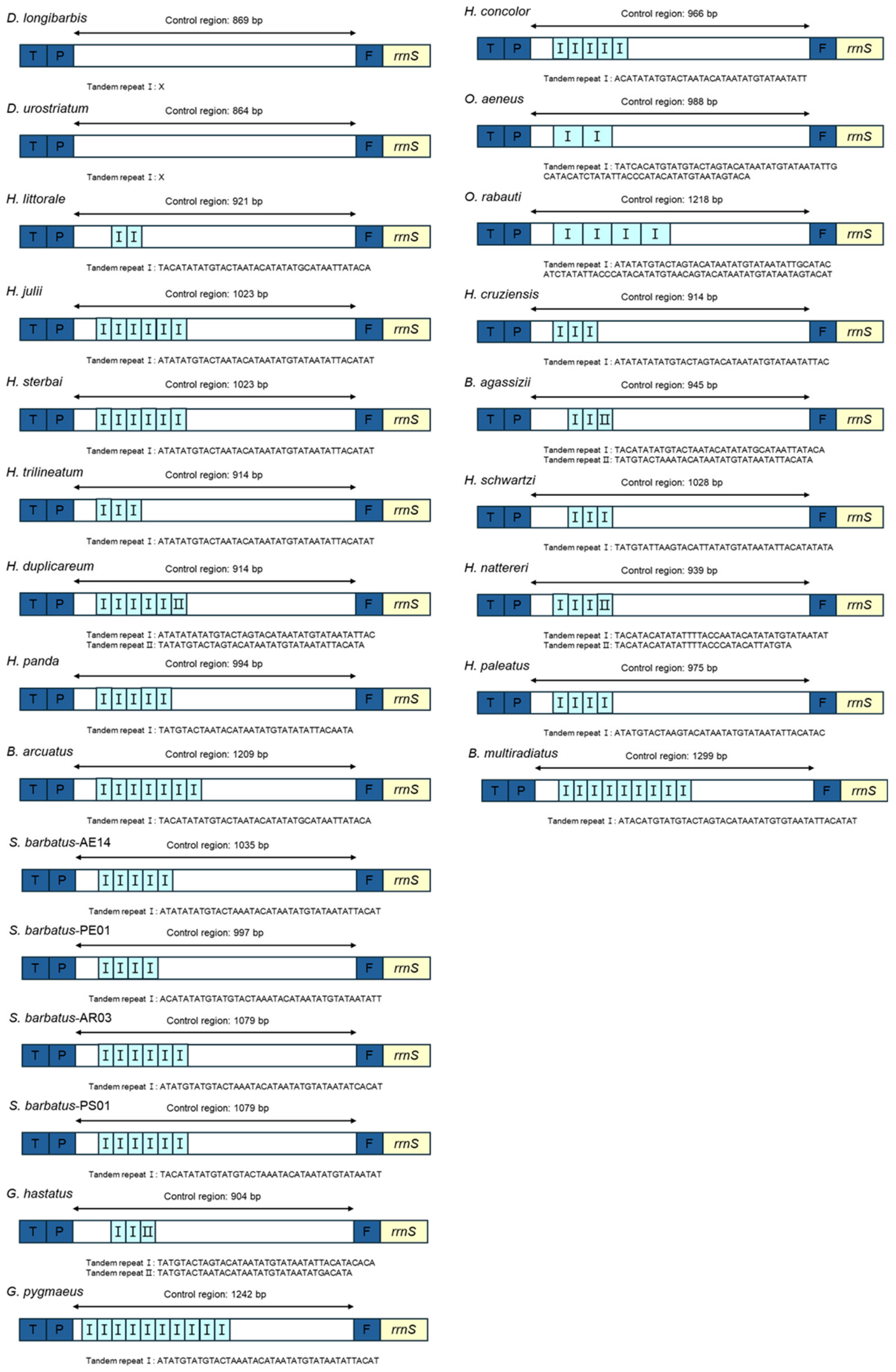

Figure S5. Tandem repeat sequences in the control regions of 24 species in the Callichthyidae family including the name of each species, the length of the control region, and the sequence of the tandem repeat. Information about each species is provided in **Table S1**.

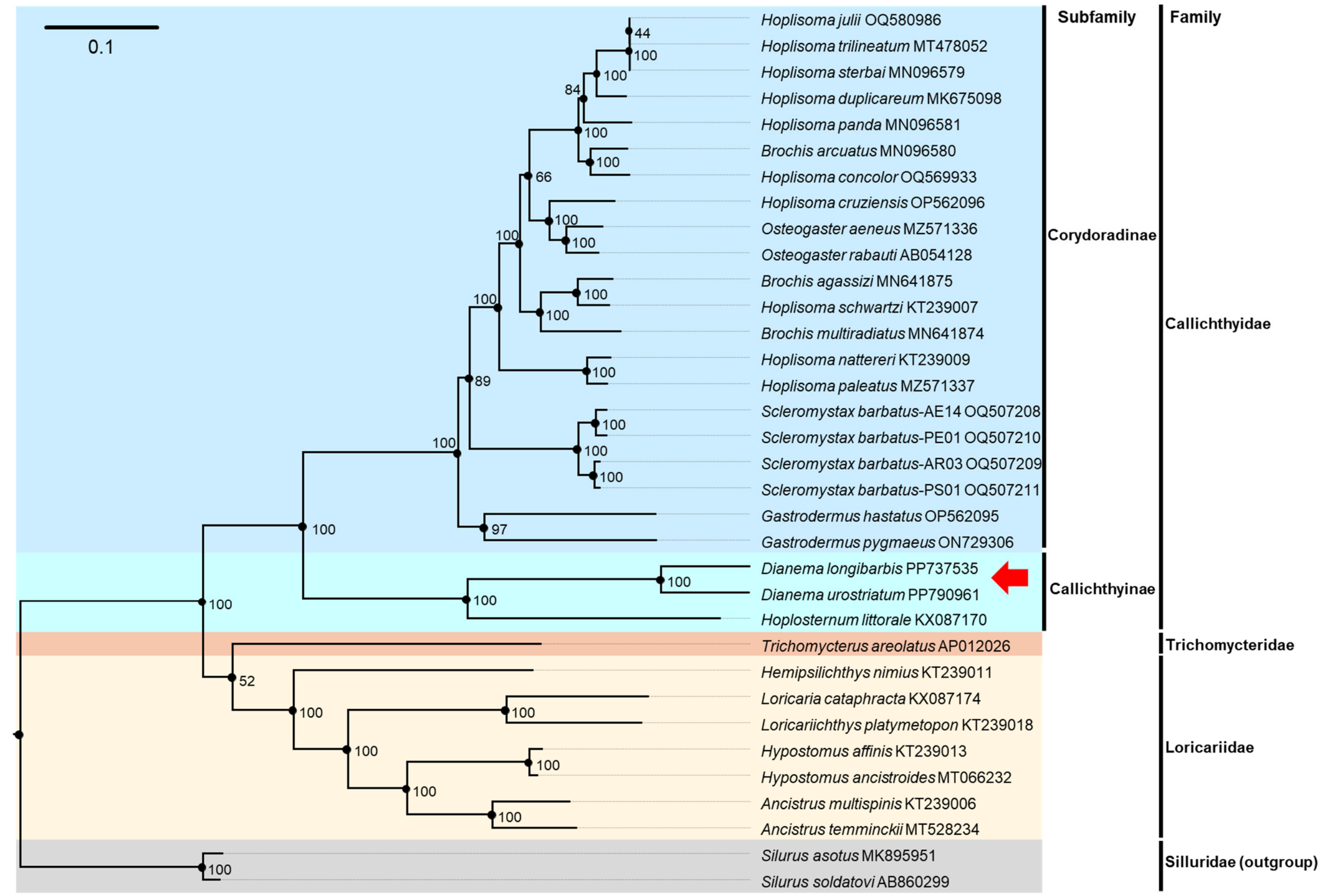

Figure S6. The phylogenetic tree of 34 published mitogenomes of Siluriformes, including mitogenomes of *D. longibarbis* and *D. urostriatum*, based on the concatenated nucleotide sequences of 13 PCGs. The numbers on the nodes indicate ML bootstrap percentages. Genbank accession numbers for published sequences are incorporated. The black arrow represents the catfish species analyzed in this study. Reference on the mitogenome data used in this analysis is appended in **Table S1**.

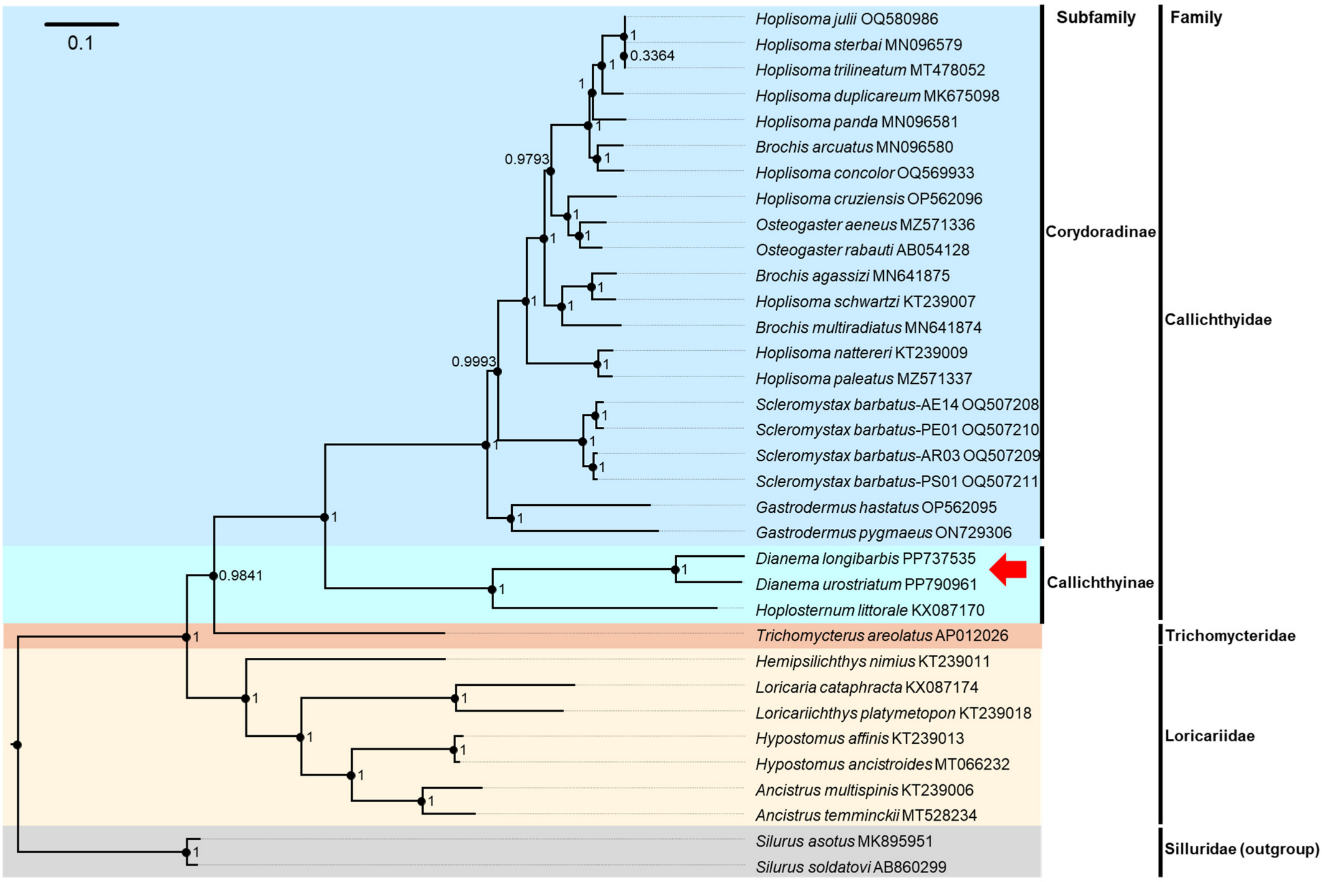

Figure S7. The phylogenetic tree of 34 published mitogenomes of Siluriformes, including mitogenomes of *D. longibarbis* and *D. urostriatum*, based on the concatenated nucleotide sequences of 13 PCGs. The numbers on the nodes indicate Bayesian posterior probability. Genbank accession numbers for published sequences are incorporated. The black arrow represents the catfish species analyzed in this study. Reference on the mitogenome data used in this analysis is appended in **Table S1**.

Table S1. The mitogenome data used in the phylogenetic analysis including information on the species, genus, and family, mitogenome length, NCBI accession number, and reference for each fish.

Family	Genus	Species	Length (bp)	Accession number	Reference
Callichthyidae	*Dianema*	*Dianema longibarbis*	16,493	PP737535	This study
	Dianema	*Dianema urostriatum*	16,495	PP790961	This study
	Hoplosternum	*Hoplosternum littorale*	16,597 (P)	KX087170	[14]
Corydoradinae	*Brochis*	*Brochis agassizii*	16,562	MN641875	[38]
	Brochis	*Brochis arcuatus*	16,822	MN096580	[40]
	Brochis	*Brochis multiradiatus*	16,916	MN641874	[51]
	Gastrodemus	*Gastrodemus hastatus*	16,518	OP562095	[45]
	Gastrodemus	*Gastrodemus pygmaeus*	16,840	ON729306	[46]
	Hoplisoma	*Holisoma julii*	16,636	OQ580986	Unpublished
	Hoplisoma	*Hoplisoma concolor*	16,579	OQ569933	[48]
	Hoplisoma	*Hoplisoma cruziensis*	16,531	OP562096	[45]
	Hoplisoma	*Hoplisoma duplicareum*	16,667	MK675098	[41]
	Hoplisoma	*Hoplisoma nattereri*	16,557	KT239009	[43]
	Hoplisoma	*Hoplisoma paleatus*	16,593	MZ571337	[38]
	Hoplisoma	*Hoplisoma panda*	16,611	MN096581	[44]
	Hoplisoma	*Hoplisoma schwartzi*	16,632	KT239007	[52]
	Hoplisoma	*Hoplisoma sterbai*	16,636	MN096579	[47]
	Hoplisoma	*Hoplisoma trilineatum*	16,526	MT478052	[42]
	Scleromystax	*Scleromystax barbatus*	16,651	OQ507208	[53]
	Scleromystax	*Scleromystax barbatus*	16,614	OQ507210	[53]
	Scleromystax	*Scleromystax barbatus*	16,694	OQ507209	[53]
	Scleromystax	*Scleromystax barbatus*	16,693	OQ507211	[53]
	Osteogaster	*Osteogaster aeneus*	16,604	MZ571336	[38]
	Osteogaster	*Osteogaster rabauti*	16,831	AB054128	[54]
Trichomycteridae	*Trichomycterus*	*Trichomycterus areolatus*	16,586 (P)	AP012026	[55]
Loricariidae	*Ancistrus*	*Ancistrus multispinis*	16,177 (P)	KT239006	[52]
	Ancistrus	*Ancistrus temminckii*	16,557	MT528234	[56]
	Hemipsilichthys	*Hemipsilichthys nimius*	16,320 (P)	KT239011	[52]
	Hypostomus	*Hypostomus affinis*	16,398 (P)	KT239013	[52]
	Hypostomus	*Hypostomus ancistroides*	16,826 (P)	MT066232	[57]
	Loricaria	*Loricaria cataphracta*	16,409 (P)	KX087174	[52]
	Loricariichthys	*Loricariichthys platymetopon*	16,262 (P)	KT239018	[52]
Siluridae	*Silurus*	*Silurus asotus*	17,385	MK895951	[58]
	Silurus	*Silurus soldatovi*	16,527	AB860299	[59]

Note: Patial mitogenomes were indicated with an abbreviation 'P'.

Table S2. Best substitution model used for phylogenetic trees.

Gene	P123/P123+2rRNA (ML, BIC)	P123/P123+2rRNA (BI, AIC)
atp6	GTR+F+I+G4/GTR+F+I+G4	GTR+F+I+G4/GTR+F+I+G4
atp8	GTR+F+I+G4/GTR+F+I+G4	GTR+F+I+G4/HKY+F+I+G4
cox1	GTR+F+I+G4/TIM2+F+I+G4	GTR+F+I+G4/GTR+F+I+G4
cox2	GTR+F+I+G4/TIM2+F+I+G4	GTR+F+I+G4/GTR+F+I+G4
cox3	GTR+F+I+G4/TIM2+F+I+G4	GTR+F+I+G4/GTR+F+I+G4
cytb	GTR+F+I+G4/GTR+F+I+G4	GTR+F+I+G4/GTR+F+I+G4
nad1	GTR+F+I+G4/GTR+F+I+G4	GTR+F+I+G4/GTR+F+I+G4
nad2	GTR+F+I+G4/GTR+F+I+G4	GTR+F+I+G4/GTR+F+I+G4
nad3	GTR+F+I+G4/GTR+F+I+G4	GTR+F+I+G4/GTR+F+I+G4
nad4	GTR+F+I+G4/GTR+F+I+G4	GTR+F+I+G4/GTR+F+I+G4
nad4l	GTR+F+I+G4/GTR+F+I+G4	GTR+F+I+G4/GTR+F+I+G4
nad5	GTR+F+I+G4/GTR+F+I+G4	GTR+F+I+G4/GTR+F+I+G4
nad6	HKY+F+I+G4/HKY+F+I+G4	HKY+F+I+G4/HKY+F+I+G4
rrnS	-/TIM2+F+I+G4	-/GTR+F+I+G4
rrnL	-/TIM2+F+I+G4	-/GTR+F+I+G4

Table S3. Details on codon count and RSCU in the *D. longibarbis* mitogenome.

Codon	Count	RSCU	Codon	Count	RSCU	Codon	Count	RSCU	Codon	Count	RSCU
UUU(F)	121	1.04	UCU(S)	42	1.05	UAU(Y)	65	1.17	UGU(C)	12	1
UUC(F)	111	0.96	UCC(S)	56	1.39	UAC(Y)	46	0.83	UGC(C)	12	1
UUA(L)	146	1.38	UCA(S)	88	2.19	UAA(*)	10	3.08	UGA(W)	115	1.89
UUG(L)	26	0.25	UCG(S)	7	0.17	UAG(*)	2	0.62	UGG(W)	7	0.11
CUU(L)	115	1.09	CCU(P)	36	0.7	CAU(H)	30	0.57	CGU(R)	12	0.63
CUC(L)	84	0.79	CCC(P)	51	0.99	CAC(H)	76	1.43	CGC(R)	14	0.74
CUA(L)	237	2.24	CCA(P)	113	2.18	CAA(Q)	93	1.86	CGA(R)	46	2.42
CUG(L)	26	0.25	CCG(P)	7	0.14	CAG(Q)	7	0.14	CGG(R)	4	0.21
AUU(I)	228	1.49	ACU(T)	44	0.58	AAU(N)	66	1.06	AGU(S)	13	0.32
AUC(I)	78	0.51	ACC(T)	107	1.41	AAC(N)	59	0.94	AGC(S)	35	0.87
AUA(M)	146	1.57	ACA(T)	149	1.97	AAA(K)	77	1.83	AGA(*)	0	0
AUG(M)	40	0.43	ACG(T)	3	0.04	AAG(K)	7	0.17	AGG(*)	1	0.31
GUU(V)	67	1.33	GCU(A)	66	0.8	GAU(D)	31	0.81	GGU(G)	44	0.75
GUC(V)	32	0.64	GCC(A)	134	1.63	GAC(D)	46	1.19	GGC(G)	70	1.2
GUA(V)	95	1.89	GCA(A)	124	1.51	GAA(E)	85	1.73	GGA(G)	98	1.68
GUG(V)	7	0.14	GCG(A)	4	0.05	GAG(E)	13	0.27	GGG(G)	22	0.38

Table S4. Details on codon count and RSCU in the *D. urostriatum* mitogenome

Codon	Count	RSCU	Codon	Count	RSCU	Codon	Count	RSCU	Codon	Count	RSCU
UUU(F)	122	1.05	UCU(S)	40	1	UAU(Y)	60	1.1	UGU(C)	7	0.58
UUC(F)	110	0.95	UCC(S)	53	1.33	UAC(Y)	49	0.9	UGC(C)	17	1.42
UUA(L)	126	1.19	UCA(S)	91	2.28	UAA(*)	10	3.08	UGA(W)	115	1.87
UUG(L)	18	0.17	UCG(S)	6	0.15	UAG(*)	2	0.62	UGG(W)	8	0.13
CUU(L)	102	0.96	CCU(P)	37	0.71	CAU(H)	35	0.64	CGU(R)	9	0.47
CUC(L)	85	0.8	CCC(P)	57	1.1	CAC(H)	75	1.36	CGC(R)	19	1
CUA(L)	278	2.63	CCA(P)	108	2.08	CAA(Q)	93	1.84	CGA(R)	38	2
CUG(L)	26	0.25	CCG(P)	6	0.12	CAG(Q)	8	0.16	CGG(R)	10	0.53
AUU(I)	220	1.46	ACU(T)	47	0.6	AAU(N)	58	0.95	AGU(S)	12	0.3
AUC(I)	82	0.54	ACC(T)	113	1.44	AAC(N)	64	1.05	AGC(S)	38	0.95
AUA(M)	140	1.54	ACA(T)	148	1.89	AAA(K)	79	1.88	AGA(*)	0	0
AUG(M)	42	0.46	ACG(T)	6	0.08	AAG(K)	5	0.12	AGG(*)	1	0.31
GUU(V)	68	1.35	GCU(A)	64	0.8	GAU(D)	36	0.92	GGU(G)	47	0.8
GUC(V)	31	0.61	GCC(A)	136	1.69	GAC(D)	42	1.08	GGC(G)	72	1.23
GUA(V)	93	1.84	GCA(A)	113	1.41	GAA(E)	83	1.69	GGA(G)	88	1.5
GUG(V)	10	0.2	GCG(A)	8	0.1	GAG(E)	15	0.31	GGG(G)	27	0.46
